# Experimental Identification of Dynamic State Boundary Surface of Red Mudstone Fill Material

**DOI:** 10.3390/ma19194178

**Published:** 2026-09-30

**Authors:** Gaofeng Pan, Xianfeng Liu, Kang Chen, Yanfei Zhang, Danxi Sun, Qihong Wu, Ran Tang

**Affiliations:** 1 School of Architecture and Civil Engineering, Chengdu University, Chengdu 610106, China; 2 Key Laboratory of High-Speed Railway Engineering of Ministry of Education, School of Civil Engineering, Southwest Jiaotong University, Chengdu 610031, China; 3 Sichuan Engineering Research Center for Mechanical Properties and Engineering Technology of Unsaturated Soils, Chengdu University, Chengdu 610106, China; 4 School of Transportation, Changsha University of Science and Technology, Changsha 410114, China; 5 School of Civil and Environmental Engineering, Zhengzhou University of Aeronautics, Zhengzhou 450046, China; 6 School of Transportation, Kashi University, Kashi 844000, China

**Keywords:** red mudstone fill material, two-stage behavior, dynamic properties, dynamic state boundary surface

## Abstract

Red mudstone fill material (RMF) has been used as a substitute fill material in railway subgrades, but its allowable dynamic response under long-term cyclic loading has not been clearly identified. This paper presents an experimental identification of the dynamic state boundary surface (DSBS) of RMF under different water contents and cyclic stress levels. A series of single-stage cyclic triaxial tests with up to 50,000 loading cycles was performed on compacted RMF specimens. The DSBS was illustrated by the isolines of loading cycles and cyclic stress in terms of permanent axial strain, strain amplitude, axial strain rate, equivalent Young’s modulus, and dissipated energy. Clear two-stage behavior was observed in the evolution of permanent deformation, equivalent Young’s modulus and dissipated energy, indicating that the effect of loading cycles should be incorporated into the state boundary framework. The DSBS based on deformation-related variables showed pronounced water content dependence, but the gradual variation in these variables led to ambiguity in identifying the shakedown ranges, especially the creep limit. In contrast, the dissipated-energy-based DSBS exhibited a stable lower boundary at high loading cycles. Two characteristic dissipated energy levels, 0.01 kPa and 0.04 kPa, were therefore defined as the boundaries for the shakedown limit and creep limit of RMF, respectively, regardless of water content and cyclic stress state. The energy classification was supported by saturated RMF tests and field excitation data from the Dazhou–Chengdu railway. These results suggest that dissipated energy can serve as a unified indicator for evaluating the stability of RMF subgrade, and that the DSBS provides a practical framework for characterizing the allowable dynamic response of clayey fill materials.

## 1. Introduction

Understanding the dynamic behavior of subgrade fill material is of great significance for ensuring the safety and smoothness of high-speed railways, for which a rigorous requirement for millimeter-level control of subgrade settlement has been adopted in the Chinese standard. The shakedown theory proposed by Werkmeister et al. [1,2] is broadly used to evaluate the long-term stability of the subgrade filled with unbound granular materials (UGMs). A quantitative criterion in terms of the magnitude of axial strain has been used in the European standard. The validity of the criterion has not been shown in clayey soils such as silt [3], silty clays [4,5], soft clays [6,7], etc., because the classification criteria for UGMs were different from the real deformation responses of clays. In contrast, the general deformation modes in the shakedown concept, i.e., plastic shakedown (range A), plastic creep (range B) and incremental collapse (range C), have been identified in both UGMs and clays.

In shakedown theory, range A indicates an ultimately stable deformation response, range B refers to an unstable deformation at a low rate with loading cycles, and range C represents the increasing permanent deformation at a high rate up to failure. It is widely observed for many geomaterials that the transition from range A to range C occurs with increasing cyclic stress [1,2,8,9,10,11]. The similarity in deformation responses implies that there is a unified framework governing the dynamic behavior of soils within an overall pattern. As challenges are still being encountered in identifying the long-term stability of the subgrade, a unified framework is a valuable tool for researchers and practitioners.

Indeed, many attempts have been made to develop a framework describing the mechanical behavior of soils. For instance, Yao et al., Wood and Maeda, and Yin et al. [12,13,14] attempted this, referring to the permissible state under different shear paths and earth structures. SBS lines are conceived within the framework of critical soil mechanics (Cam-clay model, CCM) [12]—the SBS defines the upper limit of state parameters such as stress and void ratio under monotonic static loading, serving as the fundamental criterion for evaluating the static stability of soils. A common pattern of energy dissipation proposed by García-Rojo and Herrmann [15] has been employed: the structure evolution, associated with changes in stress and strain state, can be attributed to the dissipated energy in soils. The distribution of dissipated energy in friction and volume has led to original and modified CCM [4,12], as well as other advanced models based on CCM [16,17,18]. Such ideas could also be implemented in cyclic tests through a unified framework based on dynamic state boundary surface (DSBS)—which extends the SBS concept by further incorporating loading cycles and dissipated energy to characterize the dynamic response of soils under long-term cyclic loading, and to distinguish different stability ranges (e.g., plastic shakedown, plastic creep and incremental collapse)—providing a feasible way to capture the dynamic behavior of soils. By reviewing the dynamic behaviors of different types of geomaterials [11], we have established the relation between cyclic stress and the unit dissipated energy (*E_d_*), thus allowing for the general interpretation of deformation responses. In addition, the significant dependency of structure on the evolution of *E_d_* has been shown by García-Rojo and Herrmann, Tao et al. and Qian et al. [8,9,15] through numerical analysis and experimental investigations. Their findings have verified a similar pattern between dissipated energy and cyclic stress for clays and UGMs, in other words, the presence of DSBS. In this case, theoretical methods such as the bubble model [16] and the micromechanics-based model [14,18] have been developed.

However, these models require a large number of parameters (more than ten), which makes model calibration and practical application difficult. Moreover, the dynamic response is more complex than the static mechanical behavior because a number of additional variables—including the number of loading cycles, strain amplitude, permanent axial strain and axial strain rate—must be taken into account. As indicated by Li et al. [10], although theoretical approaches have made great progress, these constitutive models do not adequately characterize behavior over a large number of loading cycles, nor do they account for the relationships among multiple dynamic responses of soils. These limitations have motivated experimental investigations into the long-term dynamic behavior of such materials. A typical example is the study by Malisetty et al. [19], who conducted cyclic tests with 50,000 loading cycles on rubber-waste granular mixtures. Following Werkmeister et al. [1,2], they found a general pattern in the axial strain and axial strain-rate responses, regardless of rubber content and confining pressure.

Given the many dynamic variables involved, dynamic behavior can be described in various ways, as demonstrated by Werkmeister et al., Tao et al. and Chen et al. [1,2,8,11], although the term DSBS was not clearly defined. Consequently, no consensus has been reached on how the DSBS should be described, leading to ambiguity in the unified characterization of the shakedown range of clayey soils. For instance, the use of different indicators for the same soil can yield markedly different classification results [9,11].

This paper presents a series of cyclic triaxial tests with 50,000 loading cycles to examine the long-term behavior of red mudstone fill material (RMF) under various water contents and cyclic stress levels. The objective of this study is to experimentally develop a unified DSBS framework for describing the dynamic responses of RMF. The feasibility of various criteria for identifying shakedown ranges has been clarified within the DSBS framework by assessing their robustness with cyclic stress and loading cycles. A stable dissipated energy level is proposed as an indicator for evaluating the long-term stability of RMF. The validity of the proposed criterion has been verified by combining the laboratory investigations of this study with those of Chen et al. [11], as well as the field tests carried out by Zhang et al. [20].

## 2. Materials and Methods

### 2.1. Specimen Preparation

The fully weathered red mudstone used in this study was obtained from Chengdu. The mineralogical composition showed that the red mudstone contains a high proportion of quartz (more than 70%) and a low proportion of smectite (2%) corresponding to low plasticity. More details of the basic properties of the red mudstone are provided in Table 1.

**Figure 1:**
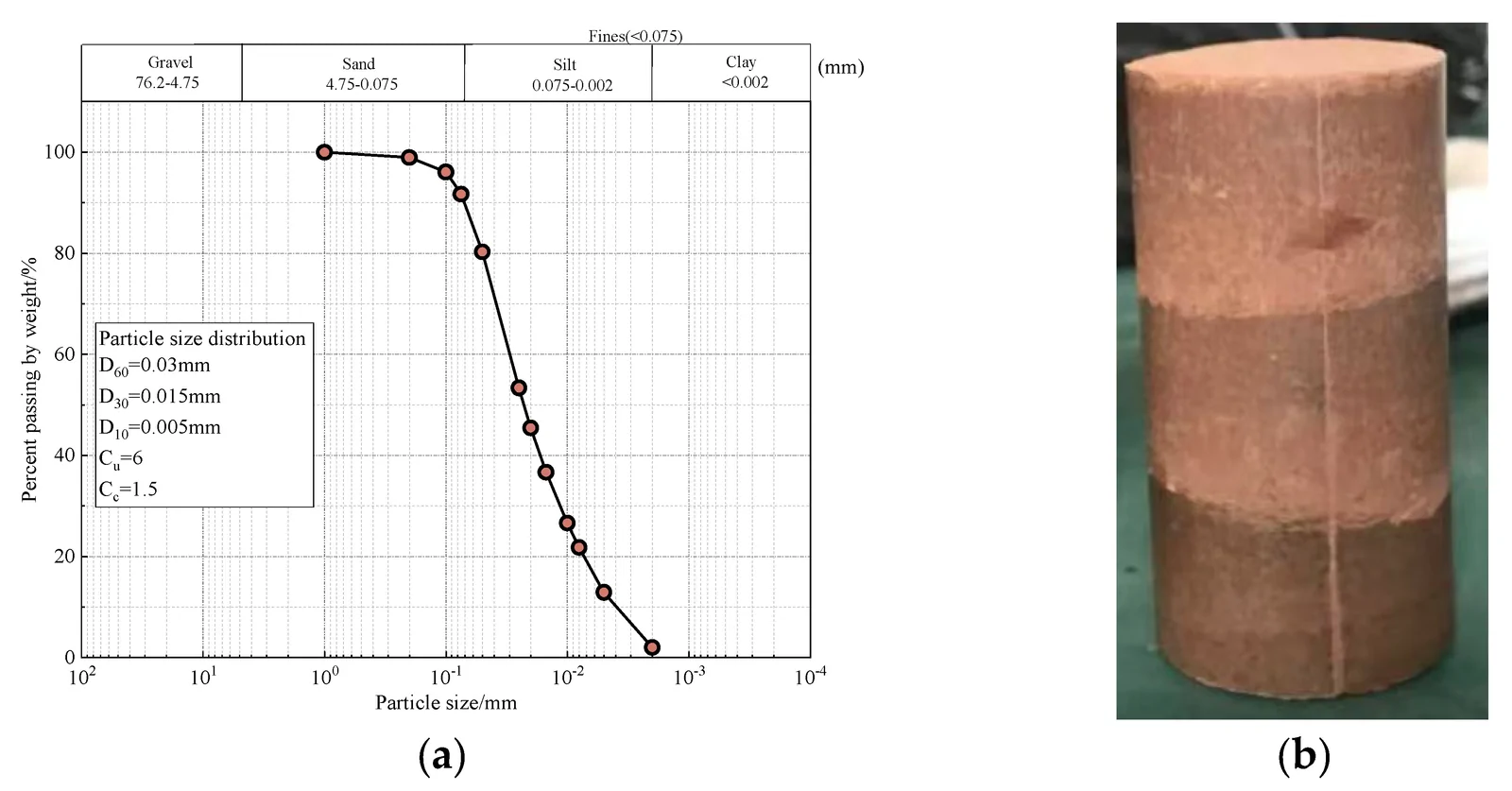
(**a**) Particle distribution of RMF; (**b**) State of the sample before experimental testing.

Fine-grained soil derived from decomposed red mudstone is preferred in subgrade construction to avoid potential deformation caused by the slaking of large blocks. Accordingly, the natural red mudstone was pre-slaked, crushed into fine particles and filtered through a 1 mm sieve. The sieved powders are mainly composed of silt (89.6%), with small fractions of clay (2.1%) and sand (8.30%) (see Figure 1), which can be classified as a silt or silty clay. The maximum dry density and optimum water content obtained from Proctor compaction tests are 2.01 g/cm^3^ and 8.7%, respectively.

Specimen preparation followed the procedure recommended by Chen et al. [21]. The RMF was oven-dried at 105 °C for 48 h and then wetted to five water contents (5%, 7%, 8.7%, 11%, 13%) by means of water spraying. The wetted soil was sealed in an airtight plastic bag for 14 days to reach water equilibrium. Cylindrical specimens with a diameter of 38 mm and a height of 76 mm were prepared by static compaction in a loading frame (see Figure 1b). An initial *e*_0_ of 0.4 (1.91 g/cm^3^ of dry density) was targeted. Such a void ratio corresponds to 95% of the maximum dry density, which conforms to the requirements of the Chinese railway design code.

### 2.2. Test Method

**Figure 2:**
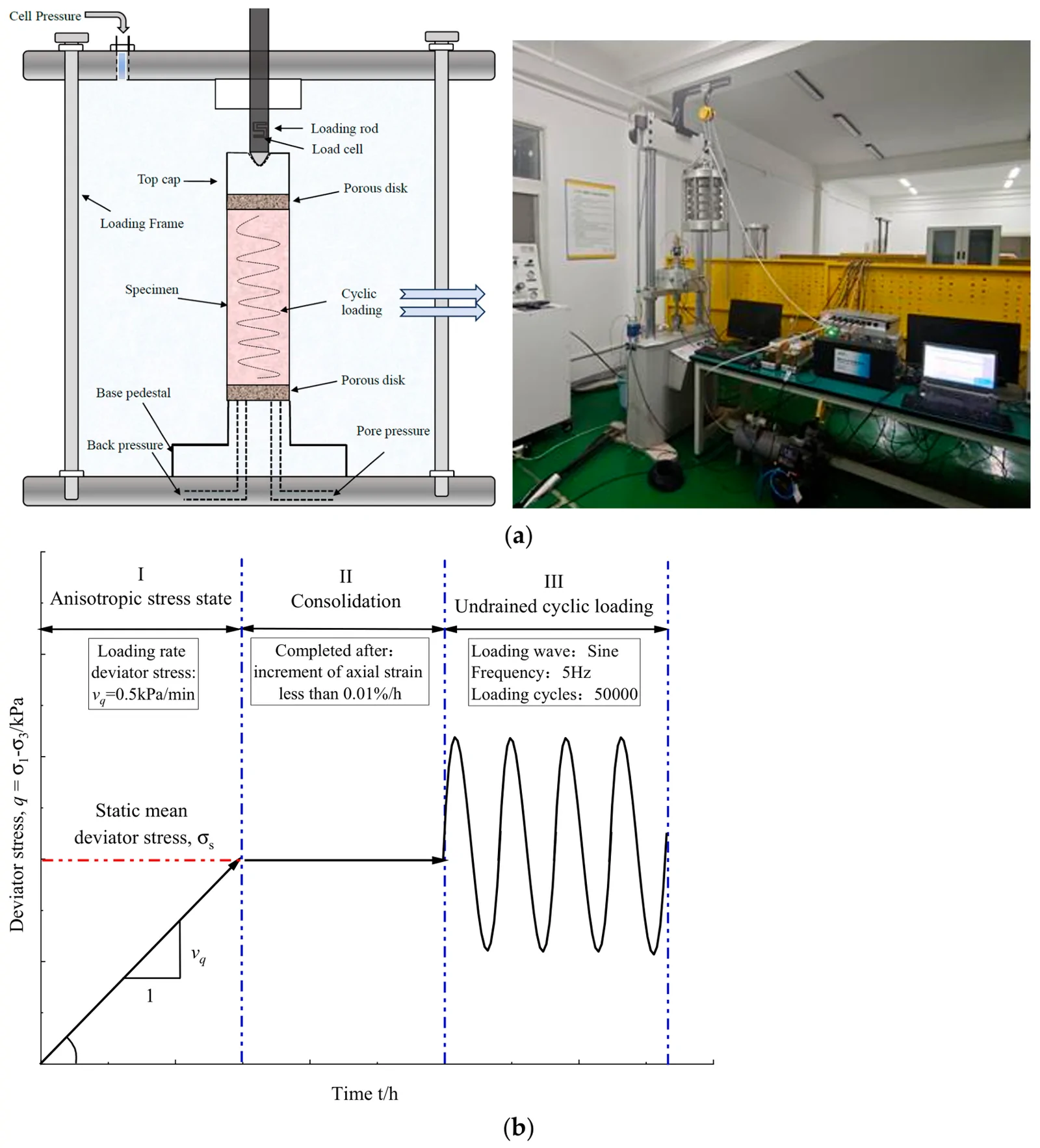
Test equipment and key sketch of loading curves: (**a**) test equipment: DYNTTS-GDS; (**b**) key sketch of loading curves.

All cyclic triaxial tests were performed using a Dynamic Triaxial Testing System (DYNTTS), produced by GDS Instruments (Hook, UK) (shown in Figure 2a). All cyclic triaxial tests were conducted in a constant temperature and humidity laboratory chamber with a temperature of 22 ± 2 °C and relative humidity of 55 ± 5%. The ambient environment was kept stable during all test procedures. The load-cell capacity, cell-pressure capacity, displacement transducer range, and loading frequency of DYNTTS are 10 kN, 2 MPa, 100 mm and 10 Hz, respectively. The cyclic stress induced by train loading significantly decreases with subgrade depth [20,22], which indicates that a higher cyclic stress ratio may occur in the upper subgrade. A confining pressure of 25 kPa was thus selected for all tests to consider the in situ stress state at low depth (around 0.6 m) of the subgrade [20,23]. The static shear strength of RMF under a confining pressure *σ*_3_ of 25 kPa was preliminarily obtained from consolidated undrained triaxial compression tests. The application of cyclic stress was based on the shear strength of RMF at each selected water content.

The single-stage loading test (SLT) recommended by Werkmeister et al., Qian et al. and Li et al. [2,9,10] was employed in this study. Figure 2b shows typical SLT loading curves. The water remaining in the drainage tube was discharged by an air pump prior to triaxial tests. The specimen was subsequently consolidated to the target anisotropic stress state, followed by uniform sinusoidal loading at a frequency of 5 Hz. As indicated by Chen et al. [23], the static mean deviator stress shown in Figure 2b reached approximately 45~55% of shear strength *q_f_*, ensuring contact between the loading bar and the top cap under large cyclic stress loading. In a field test, this setup is used to prevent the detachment of the train wheel from the railway track [23]. To attain the various shakedown responses from stable to unstable, a wide range of cyclic stress levels was applied at each water content. The complete test program was provided in Table 2. For some tests, a high cyclic stress level (e.g., 450 kPa) was imposed on dense RMF with a void ratio of 0.4 to induce the unstable deformation response. In this case, the stress states listed in Table 2 cover not only the normal subgrade range but also extended stress levels to examine a broader range of dynamic responses in the DSBS. The cyclic triaxial tests were terminated at 50,000 loading cycles or upon the specimen reaching an axial strain of 5%, whichever occurred first [11,23]. All specimens were tested under undrained conditions, given the high loading frequency and low permeability of the heavily compacted RMF. At the end of each test, the water contents of the specimens were measured, and the results showed a maximum variation of less than 0.7% relative to the initial state. This confirms the undrained condition during cyclic testing.

## 3. Results

### 3.1. General Aspects

**Figure 3:**
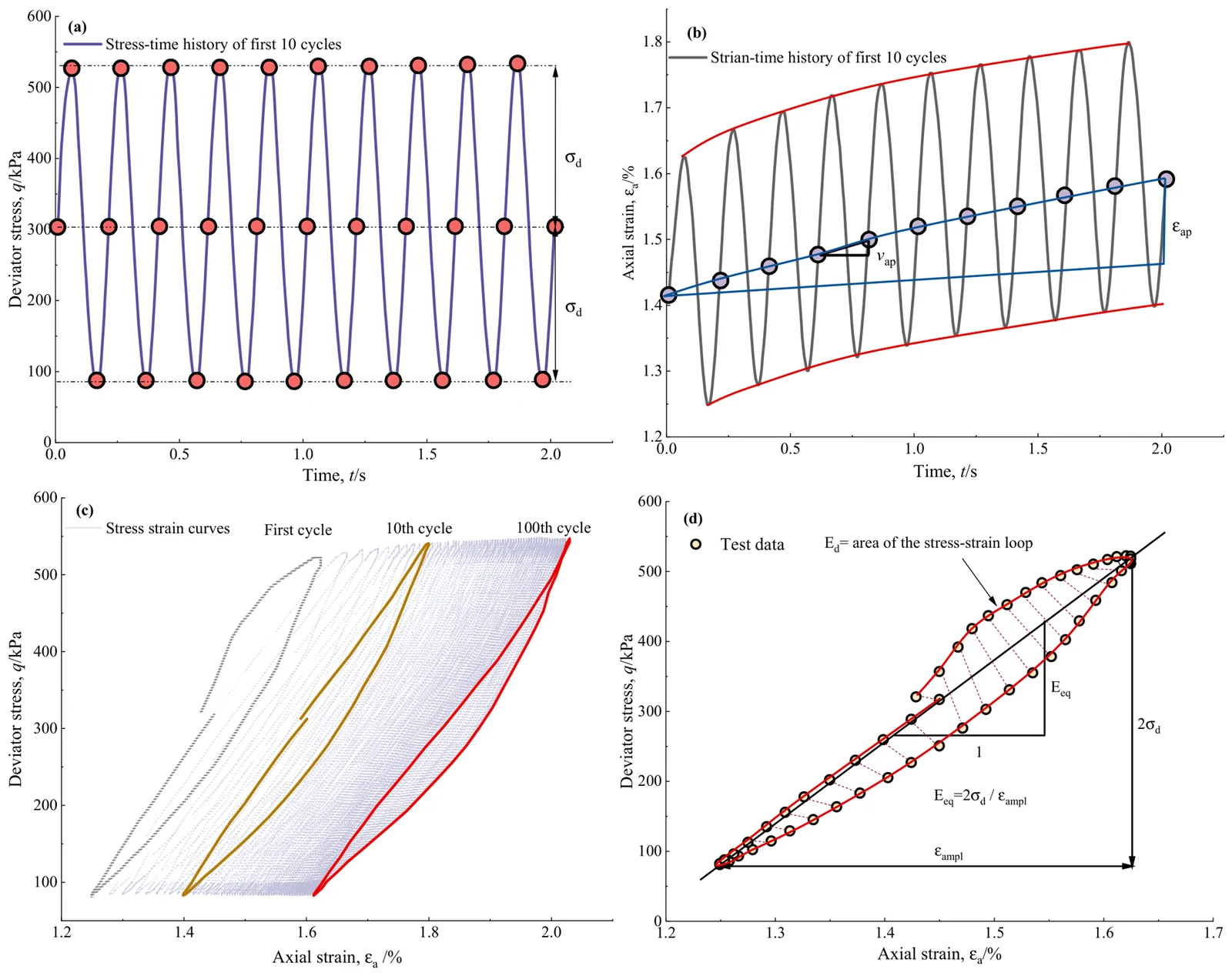
Typical cyclic triaxial test results: (**a**) stress-loading cycles for first 10 cycles; (**b**) strain-loading cycles for the first 10 cycles; (**c**) stress–strain loops for the first 100 cycles; (**d**) typical stress–strain loop used to define equivalent Young’s modulus and dissipated energy.

Following García-Rojo and Herrman, Tao et al., and Chen et al. [8,11,15], typical results of stress-loading cycles, strain-loading cycles and stress–strain loops shown in Figure 3 are employed to elaborate on the definition of dynamic properties. The cyclic stress *σ_d_* refers to the amplitude in the applied sine wave function. The strain amplitude *ε_ample_* represents the difference in axial strain in a given loading cycle. The permanent axial strain denotes the irreversible axial strain after cyclic loading. The axial strain rate is defined as the gradient of permanent axial strain with loading cycles. Then, the equivalent Young’s modulus *E_eq_* is computed as the ratio of cyclic stress to axial strain amplitude. The dissipated energy is calculated as the area enclosed by the stress–strain loop.

**Figure 4:**
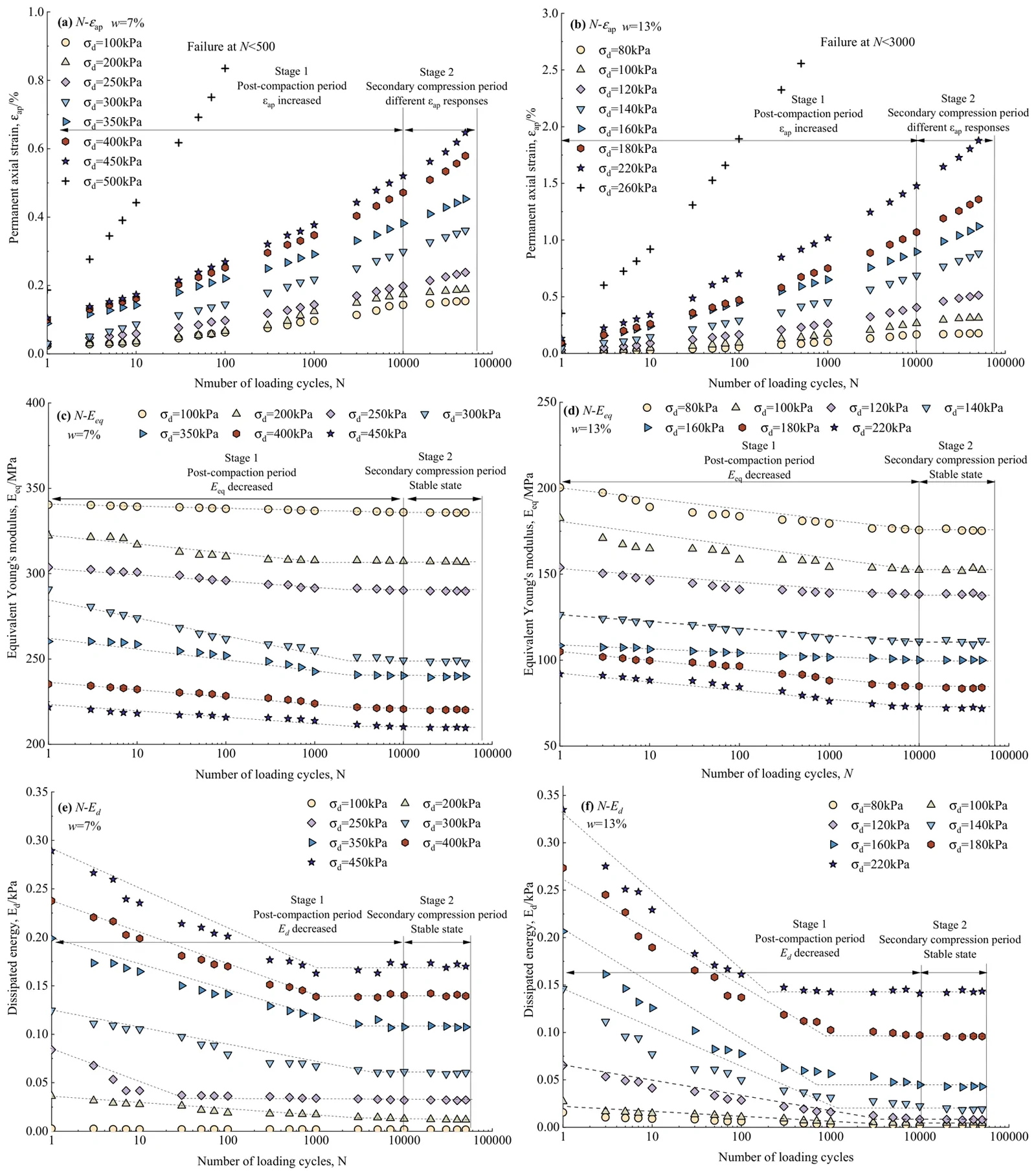
Typical illustration of two-stage behavior in permanent axial strain, equivalent Young’s modulus and dissipated energy: (**a**) permanent axial strain, w = 7%; (**b**) permanent axial strain, w = 13%; (**c**) equivalent Young’s modulus, w = 7%; (**d**) equivalent Young’s modulus, w = 13%; (**e**) dissipated energy, w = 7%; (**f**) dissipated energy, w = 13%.

As the number of loading cycles increases, the stress–strain loops move and change shape (see Figure 3c), which indicates the loading-cycle-dependent dynamic properties. Clearly, Figure 4 shows the typical two-stage behavior in permanent deformation, equivalent Young’s modulus, and dissipated energy for RMF. Stage 1, named the post-compaction period by Guo et al. [24], has been characterized by an increase in *ε_ap_* and a reduction in *E_eq_* and *E_d_* with loading cycles. Then, different deformation responses including plastic shakedown, plastic creep and incremental collapse associated with stable *E_eq_* and *E_d_* have been observed with further loading cycles (stage 2). This stage has been termed the secondary compression period by Guo et al. [24]. The representative number of loading cycles, *N*_0_, that marks the onset of stage 2 was determined to be 10,000 cycles for RMF. Such a value of *N*_0_ is much higher than values widely identified for UGMs, e.g., 1000 or 3000 cycles [2,25,26]. A larger number of loading cycles, for instance 50,000 cycles in this study, was therefore used to obtain the two-stage behavior of RMF.

**Figure 5:**
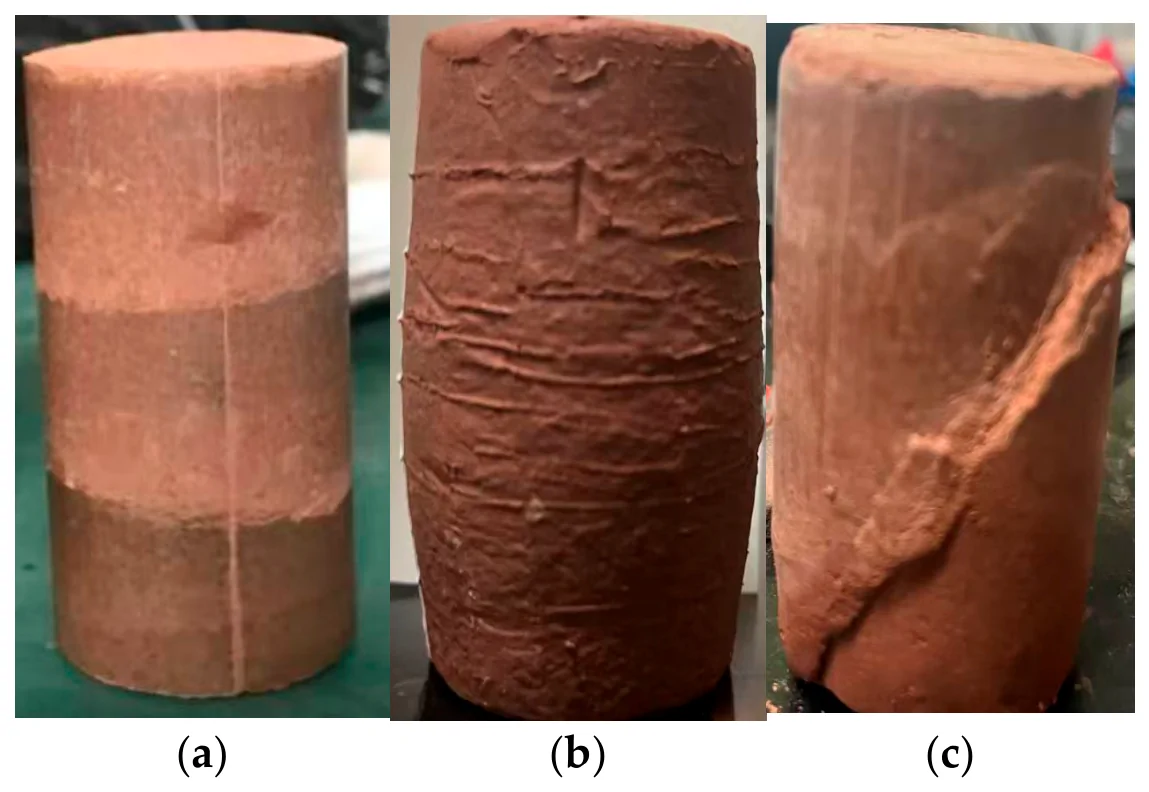
Changes in the state of the sample before and after experimental testing: (**a**) pre-experiment; (**b**) post-experiment plastic deformation and bulging; (**c**) post-experiment shear fracture.

As suggested by Chen et al. [11], stage 1 indicates the adaptation period of soil structure to cyclic stress, which results in an increase in permanent axial strain. The ultimate deformation response should be identified at stage 2 regardless of the magnitude of the permanent deformation generated at stage 1. The evolution of *E_eq_* and *E_d_* at stage 1 (Figure 4b,c) is consistent with this explanation. The reduction in *E_eq_* and *E_d_* (stage 1) confirms the significant structural change to achieve a relatively stable interlocking state in soil aggregates (stage 2) [11,15,27]. In contrast to the SBS in the static mechanical framework [12,13], the experimentally determined DSBS should include the dynamic responses at stage 1 and stage 2 to account for the structure variation induced by loading cycles. Changes in the state of the sample before and after experimental testing are shown in Figure 5.

### 3.2. DSBS for Permanent Deformation Response

**Figure 6:**
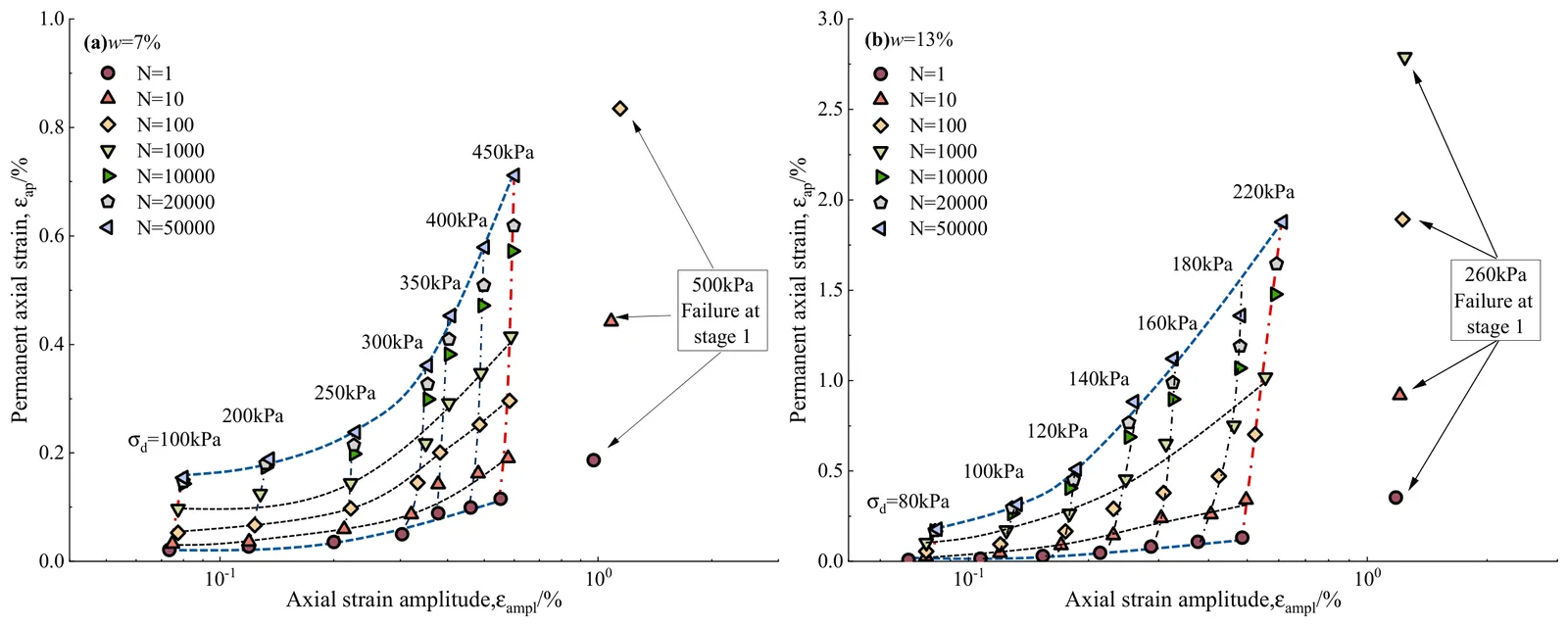
DSBS of axial strain amplitude with permanent axial strain: (**a**) permanent axial strain for first 10 cycles; (**b**) axial strain for first 10 cycles.

The idea behind the demonstration of DSBS is that the external work dissipated in the soil causes an internal response. As illustrated in Figure 3, there are various internal responses such as permanent deformation, axial strain rate, axial strain amplitude, etc. For a specific cyclic triaxial test, the internal responses are all induced by the specific cyclic load imposed on the specimen. Investigating the relationships among these responses would substantially help in understanding the dynamic behavior of RMF. Figure 6 shows typical contour plots of permanent axial strain against axial strain amplitude measured on specimens at water contents of 7% (dry side) and 13% (wet side). A state boundary surface depicted by isolines of cyclic stress (dash-dotted line) and loading cycles (dashed line) can be clearly observed. The permissible deformation response is strictly located within the DSBS; otherwise, rapid failure would be observed within a few loading cycles beyond the DSBS. For instance, under an axial strain amplitude of approximately 1%, significant permanent deformation exceeding 5% is recorded within 500 loading cycles for the specimen with a water content of 7%. As indicated by Werkmeister et al. [2], for unbound granular materials (UGMs), a straight line has been used to characterize the evolution of permanent axial strain with strain amplitude. However, the DSBS in Figure 6 is curved, indicating that the deformation response obtained by Werkmeister et al. [2] for UGMs does not coincide with that of RMF. Moreover, the shape of the DSBS varies with water content (Figure 6a,b), confirming the sensitivity of RMF’s deformation response to water content.

As shown in Figure 6, similar observations are also found in the evolution of the equivalent Yong’s modulus with permanent axial strain. Gu et al. [28] indicates that *E_eq_* is of fundamental importance to the evaluation of the permanent deformation of soils under cyclic loading. In this case, the DSBS given in Figure 6 could be used as an empirical design chart, which quantitatively describes the relation between *E_eq_* and permanent deformation. While higher *E_eq_* would result in an ultimate stable deformation response at high loading cycles, the permanent deformation of specimens with lower *E_eq_* continues to accumulate with increasing loading cycles. This condition is demonstrated in Figure 6a, i.e., for specimens under cyclic stress of 100 kPa and 450 kPa at a water content of 7%. Also, the substantial variation in deformation responses from stable to unstable is shown in a narrow band considered as the boundaries of *E_eq_*. Therefore, the precise estimation of *E_eq_* is essentially required for categorization of deformation responses. The value of *E_eq_* is assumed to be constant for conventional subgrade stability analysis, which could result in considerable miscalculation. To facilitate the assessment of long-term stability of RMF subgrade, the method of DSBS is recommended.

**Figure 7:**
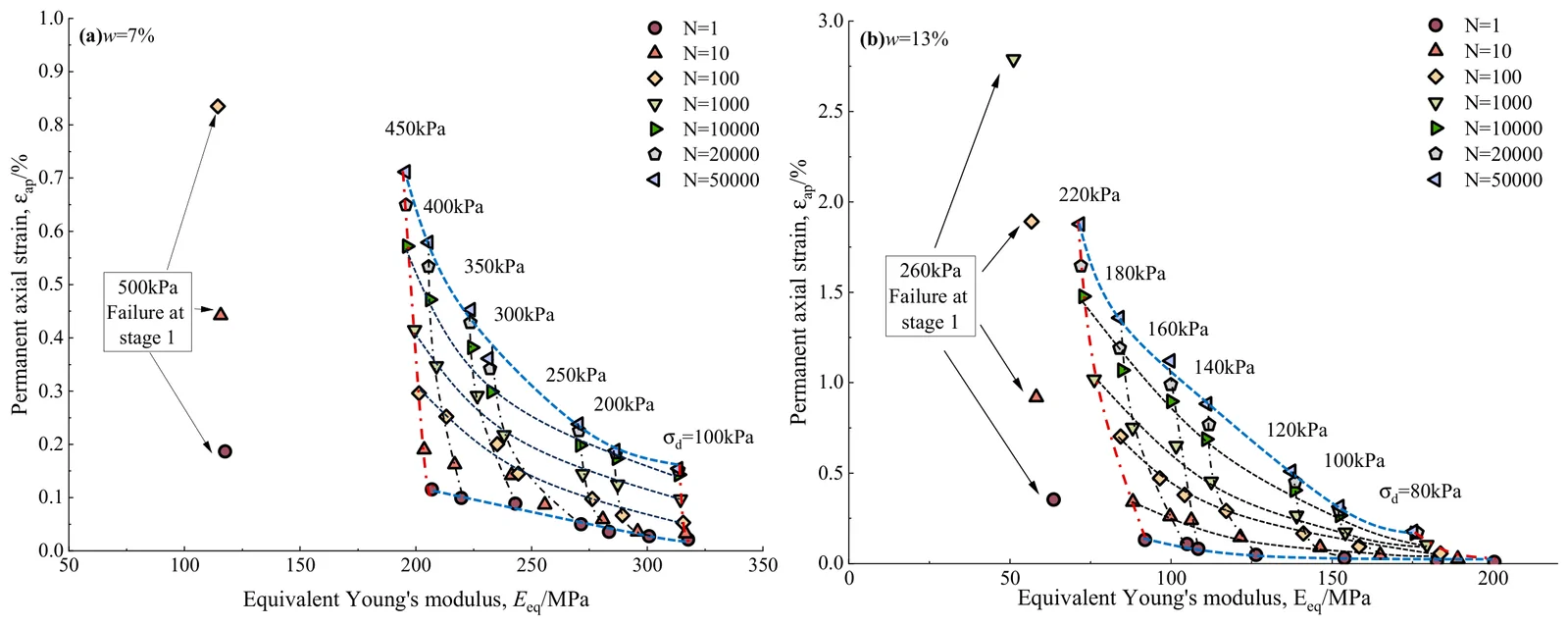
DSBS of *E_eq_* with permanent axial strain: (**a**) w = 7%; (**b**) w = 13%.

Figure 7 presents the evolution of permanent axial strain with axial strain rate, which is used as an indicator in identifying permanent deformation responses of gravels by Werkmeister et al. [1,2]. In detail, Werkmeister et al. indicate three shakedown ranges, namely plastic shakedown, plastic creep and incremental collapse. The plastic shakedown refers to an ultimate stable deformation response, characterized by a downward vertical *σ_d_* isoline, e.g., *σ_d_* = 80 kPa in Figure 7b. The plastic creep denotes the continuously accumulated deformation at a small axial strain rate. On the other hand, the incremental collapse shows a predominantly horizontal *σ_d_* isoline, which is associated with a rapid accumulation of axial strain. Consequently, two critical cyclic stress points can be defined as the shakedown limit and the creep limit, consistent with the onset of plastic creep and incremental collapse.

The gradual development observed in Figure 7 illustrates the challenge in determining the shakedown limits for RMF, especially for the creep limit. As shown in Figure 7b, a small increment of cyclic stress from 220 kPa to 260 kPa would lead to failure of the specimen, although the *ε_ap_* – *v_ap_* curves have similar shapes. Particle breakage is generally considered to induce structural modification in UGMs [29,30], while deformation of clays can be attributed to the degradation of soil aggregates [18,31]. Comparing the results of UGMs and clays, it is evident that the visual grading method proposed by Werkmeister et al. [2] for UGMs is inapplicable to silts and clays due to the different deformation mechanisms of UGMs and clayey soils. This concern is also indicated by Qian et al. [9].

**Figure 8:**
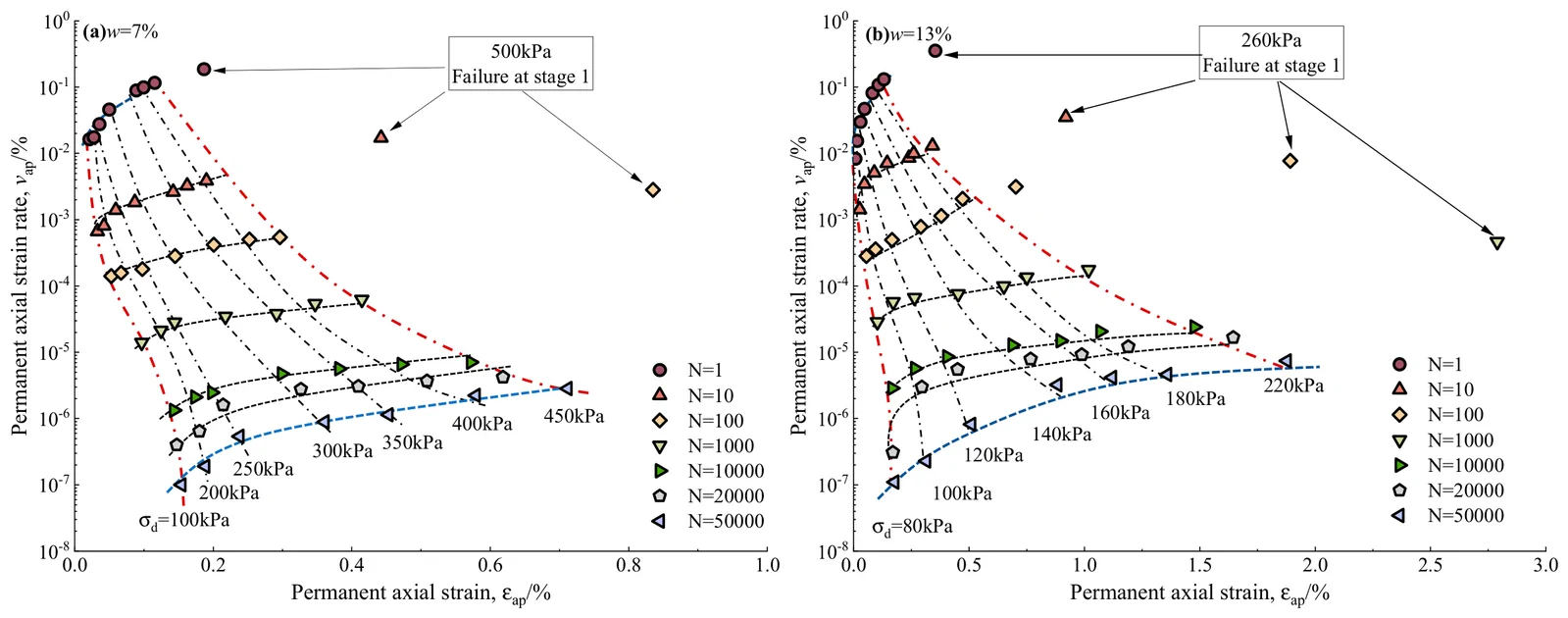
DSBS of permanent axial strain with axial strain rate: (**a**) w = 7%; (**b**) w = 13%.

Figure 8 presents the DSBS of cyclic stress with permanent axial strain. Since larger axial strain has been induced by higher cyclic stress, Gu et al. and Perez et al. [28,32] have determined the creep limit as the turning point marking a significant increase in permanent axial strain. In this case, the effect of loading cycles could be neglected, which would lead to ambiguity in the estimation of the creep limit. For instance, as shown in Figure 8a, a difference of more than 100 kPa is observed for the creep limit, determined from *N* = 1 and *N* = 50,000. Moreover, for specimens with a water content of 13% (Figure 8b), the difference in the creep limit is evident even between *N* = 20,000 and *N* = 50,000. For all tests, the error obtained from various loading cycles has been illustrated in the DSBS of permanent axial strain with cyclic stress. As can be seen from Figure 5, Figure 6, Figure 7 and Figure 8, unlike for UGMs, the validity of employing permanent deformation to characterize the shakedown behavior of clays has not been established. The source of this ambiguity is discernable: shakedown behavior represents a categorization of the permanent deformation response. Thus, the accurate identification of shakedown ranges cannot be expected to occur independently of their own response.

### 3.3. DSBS for Dissipated Energy Response

**Figure 9:**
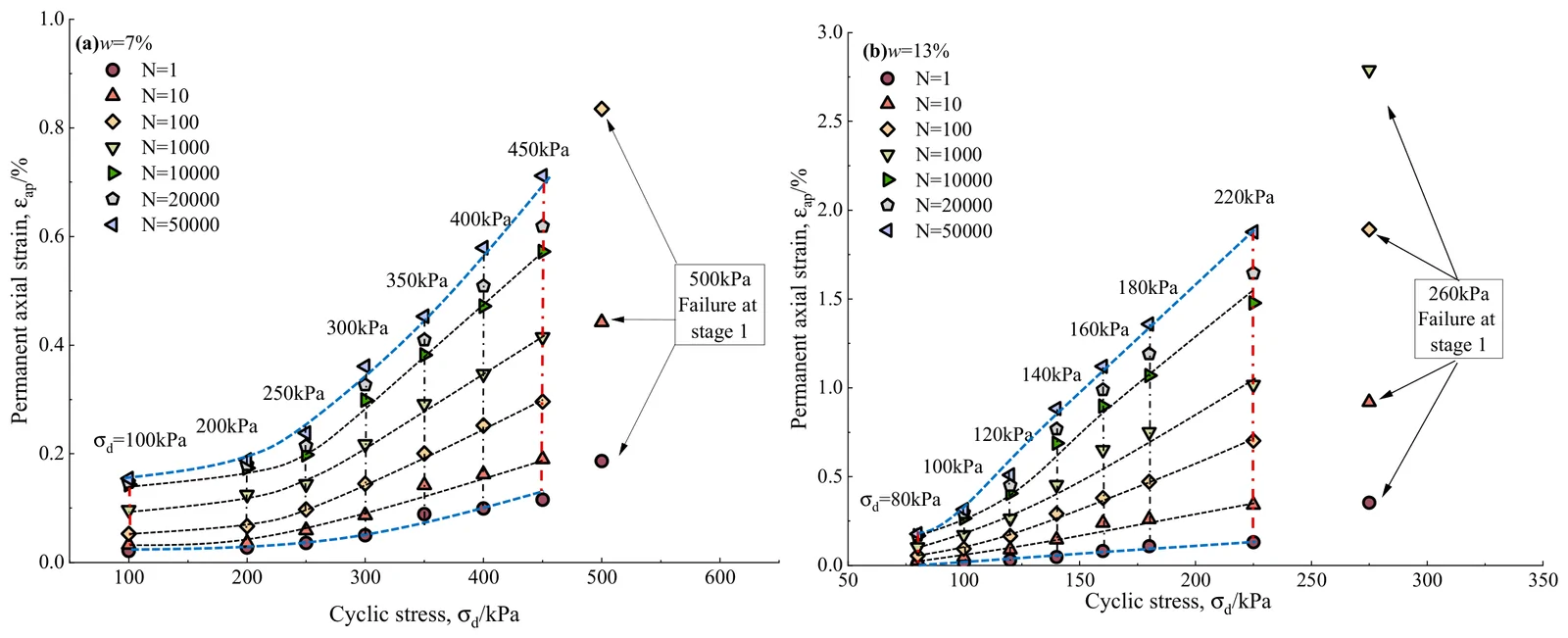
DSBS of permanent axial strain with cyclic stress: (**a**) w = 7%; (**b**) w = 13%.

It is widely recognized that the deformation of clays can be attributed to the structural adjustment under cyclic loading [18,31,33]. As indicated by García-Rojo and Herrmann [15], structural adjustment is primarily dependent on the level of unit dissipated energy absorbed in soil. Investigating the DSBS of the energy response offers a more effective way to understand the framework for shakedown analysis. Figure 9 shows the DSBS of dissipated energy with axial strain amplitude. Although the shape of the DSBS differs with water content, an increasing trend in dissipated energy with axial strain amplitude is observed for all tests. As the magnitude of *E_d_* approaches 1 kPa, the specimens experienced significant structural modifications, leading to rapid failure within a few loading cycles. Evidence of this condition can be seen from *σ_d_* = 500 kPa and 260 kPa for water contents of 7% and 13%, respectively. In addition, the dissipated energy level depends on cyclic stress, i.e., the higher the cyclic stress applied to the specimen, the greater the dissipated energy generated in RMF. Thus *E_d_* can be used as an indicator to bridge the external cyclic load and internal deformation responses.

**Figure 10:**
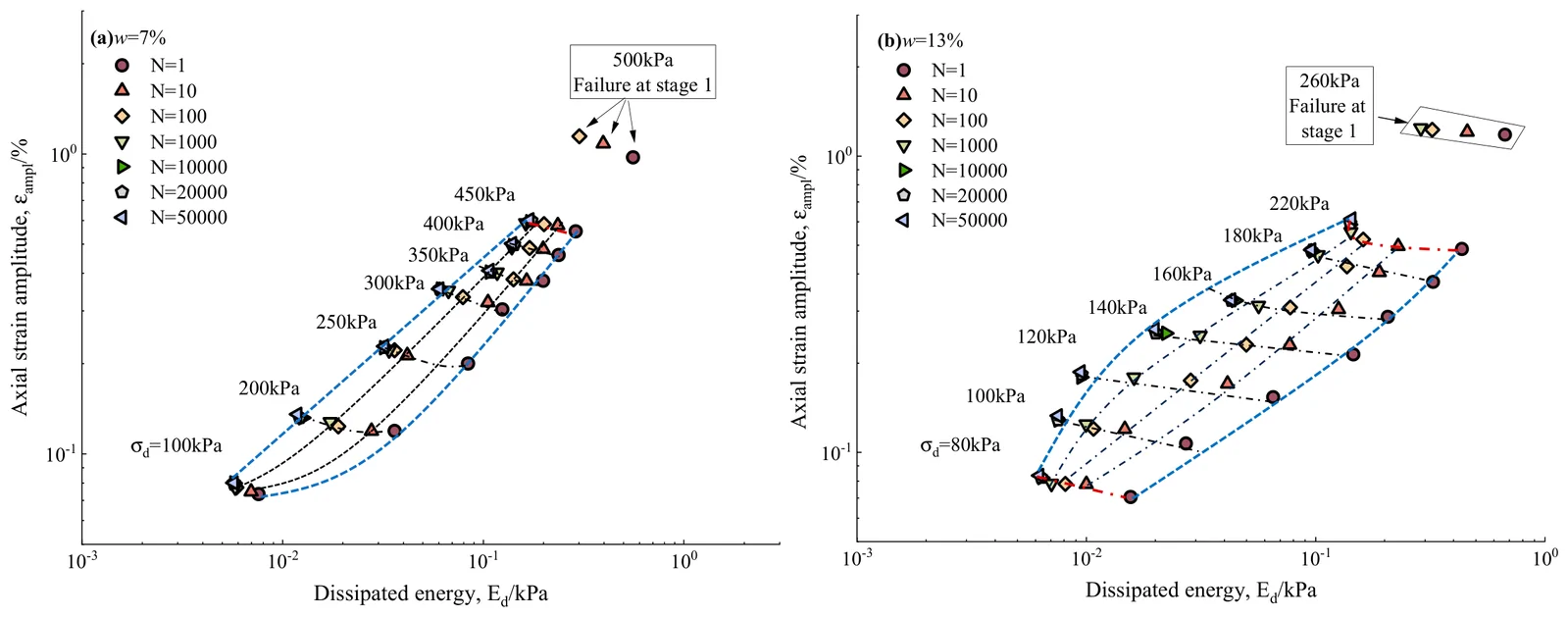
DSBS of dissipated energy with axial strain amplitude: (**a**) w = 7%; (**b**) w = 13%.

Figure 10 presents the DSBS of dissipated energy against permanent axial strain. A general trend is observed; the permanent axial strain increases with dissipated energy, which supports the observation of Figure 9. Different deformation responses correspond to different levels of dissipated energy. An explanation of this development lies within the framework of DSBS. As mentioned, all possible states shown in RMF such as range A, range B and range C must be included in DSBS. For loading cycles beyond 10,000, the dissipated energy has reached a generally stable state with slight variation. This can be verified by the approximately vertical trend of the loading-cycle isoline at *N* > 10,000 for all cyclic stress levels. As for small *E_d_* less than 10^−2^ kPa at *N* > 10,000, a stable deformation response has been identified, e.g., *σ_d_* = 100 kPa in Figure 10a. Continuous increases in *ε_ap_* were recorded in case of high dissipated-energy levels. Compared to the permanent axial strain at *N* = 20,000 and 50,000, the difference can be around 2.7 times for cyclic stress of 200 kPa and 400 kPa (see Figure 10a). As reported by García-Rojo and Herrmann [15], the small amount of dissipated energy was mainly absorbed by slight friction mechanisms without significant influence on the overall structure. In this case, the specimen reached a largely resilient response, i.e., range A has been identified. The increase in dissipated energy would induce more severe structural evolution. Continuous yielding occurred in soil, resulting in a significant accumulation of plastic strain (range B or C). As shown in Figure 10, the ultimate deformation response is dependent on the stable energy at high loading cycles. This indicates that the use of *E_d_* to characterize the shakedown behavior of RMF can minimize the effect of loading cycles.

**Figure 11:**
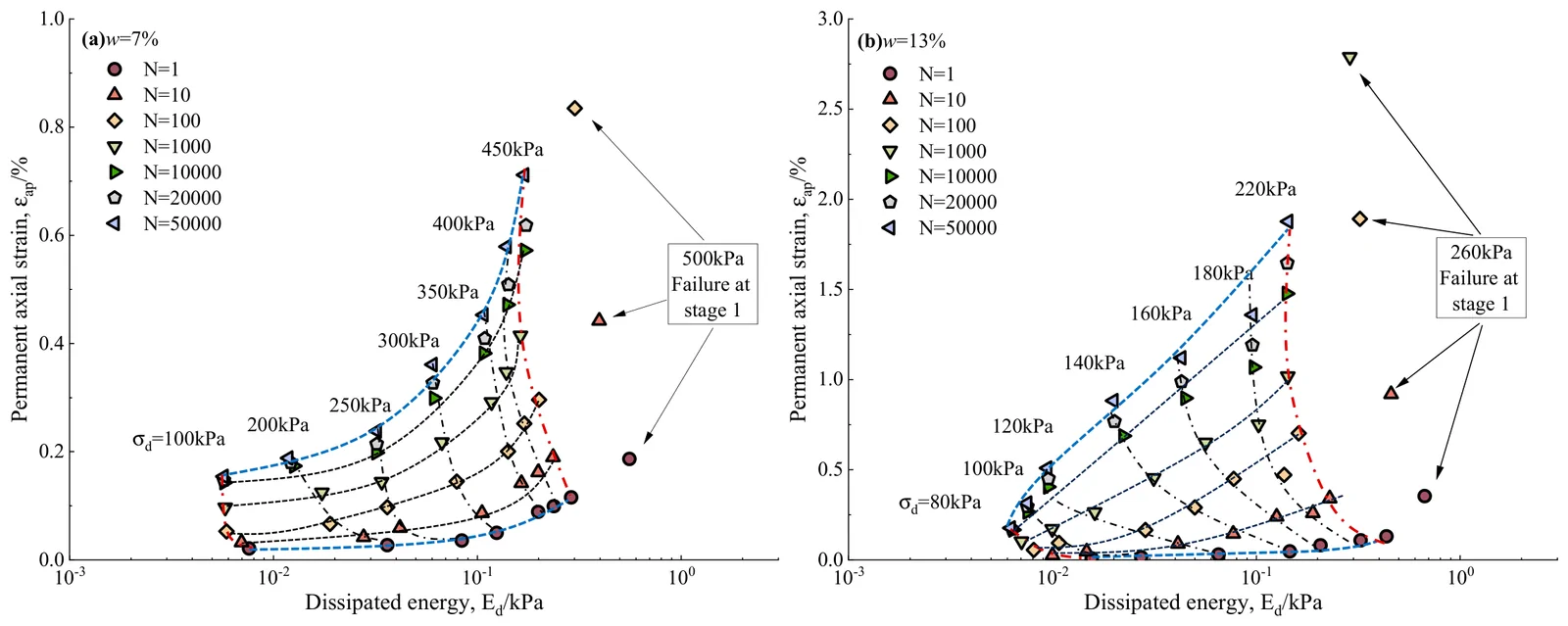
DSBS of dissipated energy with permanent axial strain: (**a**) w = 7%; (**b**) w = 13%.

Figure 11 shows the DSBS of dissipated energy with axial strain rate. Tao et al. [8] have graded the shakedown ranges by the shape of *E_d_-v_ap_* curves. They considered the non-linear decrease in *E_d_-v_ap_* curve as the unstable deformation response, i.e., range B or range C. Moreover, the largely dominant nonlinear trend is identified in range C. As shown in Figure 11, this method does not apply well to RMF, due to ambiguity in the identification of deformation responses from the curves themselves. Although for specific loading cycles, the value of axial strain is highly dependent on the magnitude of dissipated energy, the gradual change in the shape of the *E_d_-v_ap_* curve still raises the problem in determining a clear creep limit of RMF. Data presented in Figure 5, Figure 6, Figure 7, Figure 8, Figure 9, Figure 10 and Figure 11 show that the internal soil responses denote the long-term stability of RMF. These developments can be clearly observed in their respective DSBSs. However, the dynamic properties such as permanent deformation and axial strain rate should not be used to assess such behavior to avoid misunderstanding of shakedown limits. Considering the steady transfer of energy from the moving train to the subgrade fill material, the external load generating the internal response could be identified as the key factor for characterizing the shakedown ranges of RMF.

**Figure 12:**
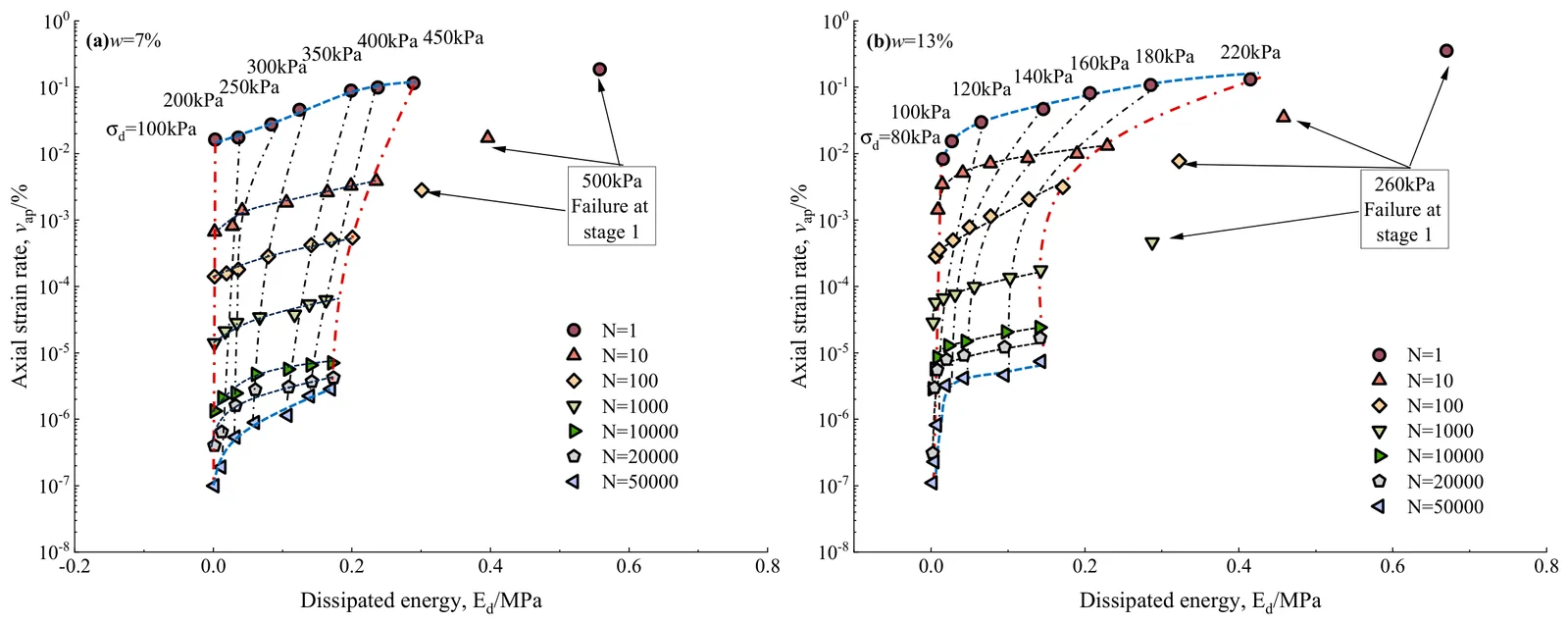
DSBS of dissipated energy with axial strain rate: (**a**) w = 7%; (**b**) w = 13%.

Figure 12 shows the evolution of cyclic stress with dissipated energy. Under a given cyclic stress, the dissipated energy reduced from an initially high level to a stable state during the secondary compression period. This indicates a unified lower boundary (blue dashed line illustrated in Figure 12) for all tests. A critical cyclic stress marking a significant increase in dissipated energy has been clearly observed in Figure 12. Based on the analysis in Figure 10, this critical cyclic stress could correspond to the noticeable change in deformation response. Therefore, Chen et al. [11] have determined the critical cyclic stress as the creep limit for clayey soils. Also, as reported by Chen et al. [11,23], a similar trend has been found in various types of geomaterials, for instance, gravels [2,34], sands [15,35], rockfills [8], silts [10,36], soft clays [7,9], etc., even though the representative numbers of loading cycles are different among these materials.

For silts and clays in particular [7,9,10], the incorporation of the DSBS into dynamic analysis provides a much more precise estimate of shakedown limits compared with conventionally used criteria, such as those proposed by Werkmeister et al. and Tao et al. [2,8]. Note that the shakedown limit for clays is only around 60% of their static shear strength [23]. The overestimation of the shakedown limit in subgrade design could be catastrophic, with unacceptable economic losses and a threat to operational safety. Therefore, an adequate investigation of the DSBS is necessary for clay and silt fill materials to avoid the progressive failure of the subgrade or even the entire railway line.

## 4. Discussion

**Figure 13:**
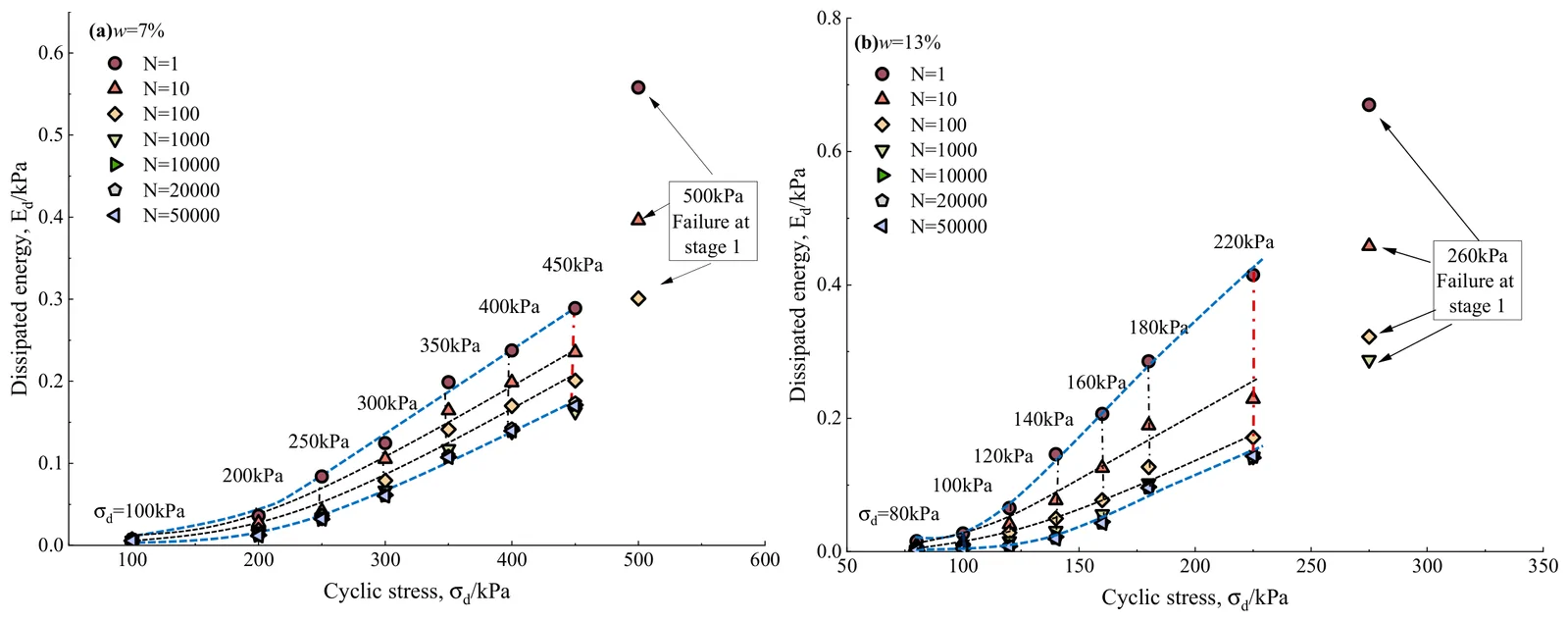
DSBS of cyclic stress with dissipated energy: (**a**) w = 7%; (**b**) w = 13%.

Figure 13 summarizes the dissipated energy at the shakedown limit *E_dlA_* and the creep limit *E_dlB_* determined from the DSBS for all water contents. Data from laboratory cyclic triaxial tests on saturated RMF obtained from Chen et al. [21,23] and field excitation tests on the Dazhou–Chengdu railway presented by Zhang et al. [20] have also been included. Two characteristic levels of the dissipated energy, *E_d_* = 0.01 kPa and *E_d_* = 0.04 kPa, are defined as the boundaries for the shakedown and creep limits of RMF across a wide range of water contents. There is insignificant variation in both *E_dlA_* and *E_dlB_*, which verifies the robustness of the energy boundaries marked by the red and blue solid lines shown in Figure 13. Note that the magnitude of imposed cyclic stress and the internal deformation vary substantially with water content. This implies the dissipated energy can be used as a unified indicator for preliminary examination of the stability of RMF subgrade. For instance, the unit dissipated energy in subgrade obtained from field test under excitation of 18- and 25-ton train load, are below 0.01 kPa and are associated with a stable subgrade settlement response, as shown by Zhang et al. [20].

Although the application of the DSBS in engineering practice has been corroborated, the limitations of the experimentally determined DSBS should also be clarified. The first limitation is the elastic boundary. Chen et al. [11] have proposed the idea that when the amount of energy absorbed by the soil is sufficiently small, only the resilient axial strain can be anticipated, i.e., pure elastic response would be observed. Such a low level of energy refers to strain amplitude below 10^−4^ for densely compacted silt [36], which is below the measurement accuracy of the test equipment used in this study. In this case, only the plastic region is plotted in all RMF DSBSs. The linear elasticity theory provides a general estimate of the elastic boundary of cyclic stress and energy. If the critical strain amplitude inducing the permanent axial strain is assumed to be 10^−4^, one obtains [11]:

(1) *σ_de_* = *E_max_ ε_ample_*

(2) $$E_{de}\;=\;\frac{1}{2}E_{max}\;{\varepsilon_{ample}}^{2}$$

where *σ_de_* is the cyclic stress at the elastic boundary, *E_de_* is the energy at the elastic boundary, *E_max_* is the elastic Young’s modulus, *ε_ample_* is the critical strain amplitude. The values of *E_max_* of RMF at water contents of 7% and 13% measured using a bender-extender element are approximately 400 MPa and 240 MPa, respectively [37]. Then, *σ_de_* and *E_de_* can be calculated from Equations (1) and (2).

**Figure 14:**
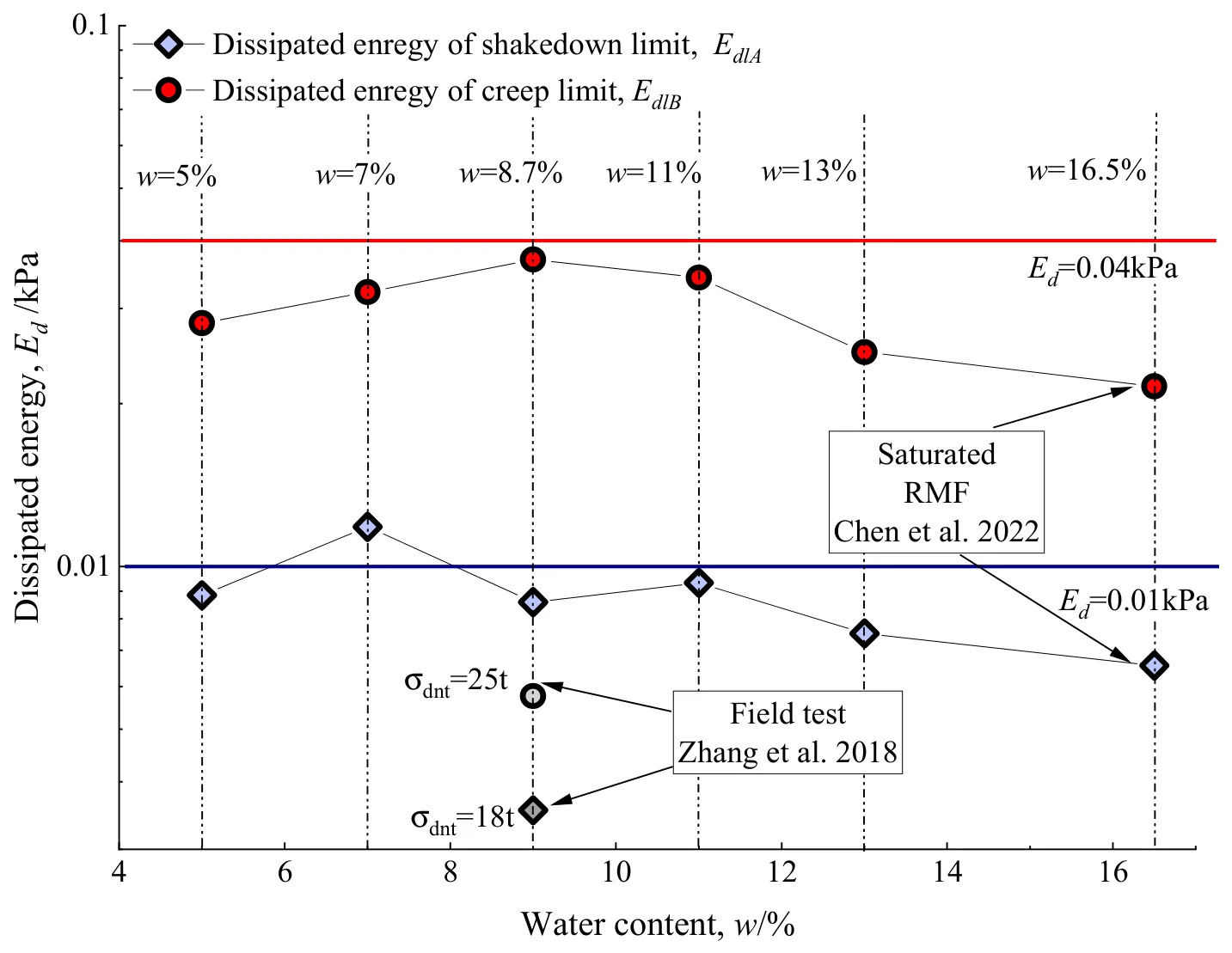
Dissipated energy of shakedown limit and creep limit [20,21].

**Figure 15:**
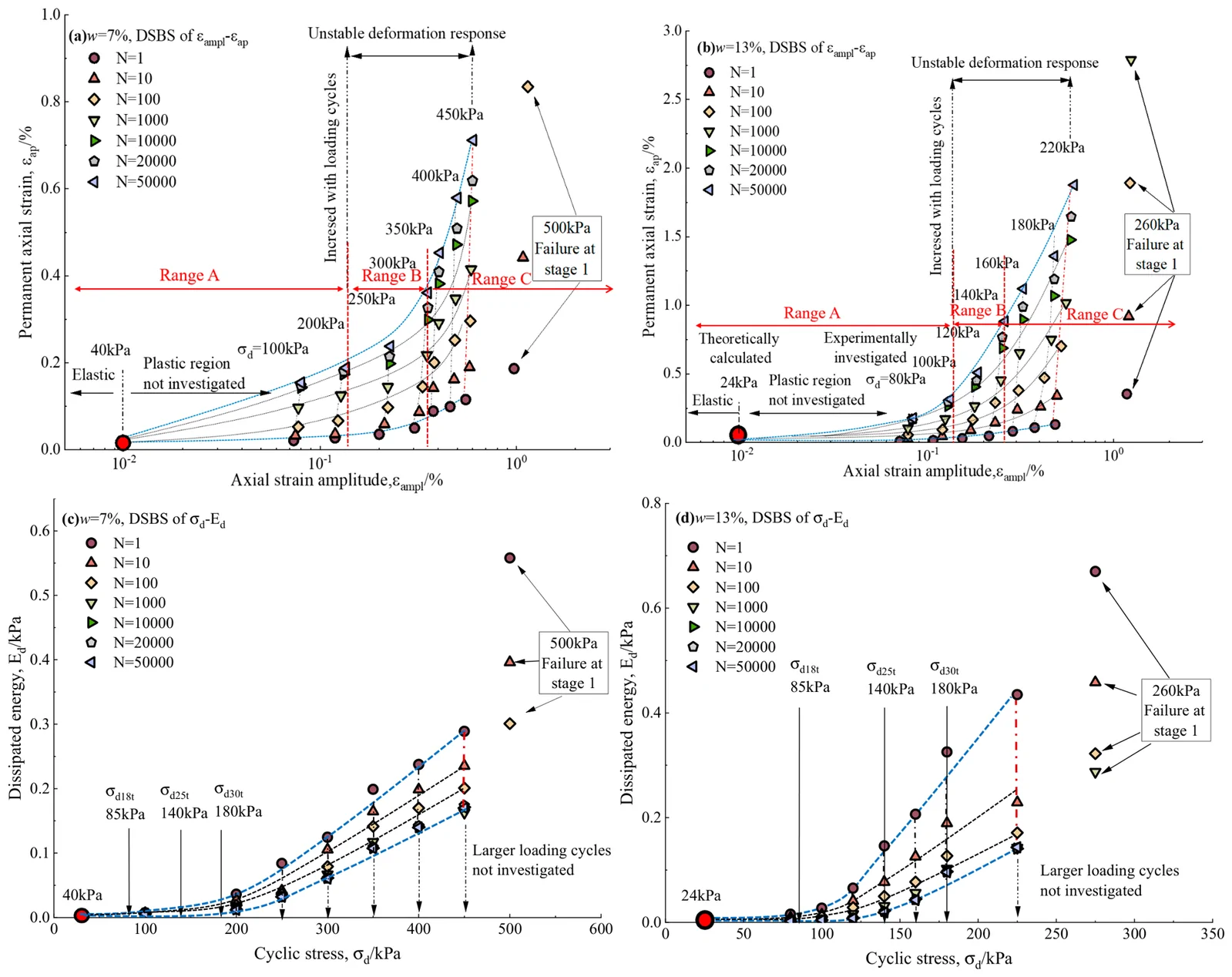
Typical illustration of DSBS with increased elastic boundary: (**a**) Permanent axial strain, w = 7%; (**b**) Permanent axial strain, w = 13%; (**c**) Dissipated energy, w = 7%; (**d**) Dissipated energy, w = 13%.

As an example, data from Figure 5 and Figure 12 are replotted in Figure 14 with the supplementation of the elastic boundary. In Figure 13, the symbol *σ_dnt_* indicates the maximum cyclic stress generated in the top layer of the subgrade, induced by the specific train axis load. For instance, *σ_dnt_* denotes the maximum cyclic stress transferred from the moving train with an 18-ton axis load. The value of *σ_dnt_* is obtained from the numerical simulation carried out by Bian et al. [22]. According to the Chinese railway design code, three representative train axis loads, 18 t, 25 t and 30 t have been selected as reference values to identify the possible state within the DSBS of RMF. As shown in Figure 14, there is only a relatively narrow distance from *σ_de_* to the tested cyclic stress, in line with the strain amplitude from 1 × 10^−4^ to around 7 × 10^−3^. The tested cyclic stress should be carefully selected to ensure that the cyclic stress state in the plastic region which is not experimentally investigated lies within range A (Figure 15c,d). In subgrade design, the deformation response in range A is preferred because long-term stability of the subgrade can be achieved. More attention should be paid to unstable deformation responses, i.e., range B and range C. For instance, the train axis loads larger than 25 t could cause a continuously developing settlement in RMF subgrade with a water content of 13%. As mentioned, the DSBS also encompasses the unstable deformation response.

To improve the theoretical basis of the DSBS framework, one valuable direction for future work is the accurate determination of the elastic boundary. Although the linear elasticity theory has been employed in this study, its validity has not been justified universally under other conditions. The results of cyclic triaxial tests obtained from Vucetic and Mortezaie and Vucetic [38,39] indicate that the cyclic stress–strain behavior in the elastic region could also be hysteretic due to a viscous effect. Essentially nonlinear elastic behavior is observed, which would lead to an overestimation of the elastic stiffness and energy. Incorporating nonlinear dynamic elasticity theory into the DSBS would be beneficial.

Another consideration is the number of loading cycles. In Figure 15a,b, the permanent deformation in range B and range C would increase with loading cycles, which implies the vertical shift of the upper boundary of *ε_ample_* − *ε_ap_* DSBS as more loading cycles are applied. However, the loading cycles cannot be infinite in a realistic test. As shown in Figure 7, under the cyclic stress of 350 kPa, the axial strain rate for a specimen with a water content of 7% is close to 10^−6^% per cycle at *N* = 50,000. In this case, a 1% increase in permanent axial strain requires more than one million loading cycles, and the test could take more than 2 months to perform. Theoretical and empirical models linking the permanent axial strain with loading cycles, such as those proposed by Werkmeister et al., Gu et al. and Li et al. [2,10,28], would potentially extend the DSBS framework for deformation responses.

On the other hand, the two-stage behavior is clearly identified in the evolution of deformation and energy over the 50,000 loading cycles of the cyclic triaxial tests. Indeed, in some cases, larger numbers of loading cycles, e.g., 80,000 and 100,000 cycles, were also imposed on RMF specimens, and the two-stage behavior was still recorded [11,23]. The possible existence of additional stages or periods cannot be ruled out, even when the number of loading cycles is much larger than the typical value, i.e., 10,000 cycles used by Chen C. et al., Gu et al. and Qian et al. [3,9,28]. Zhalehjoo [26] presents an extensive comparison for various types of UGMs. They likened the permanent deformation under a constant cyclic stress to the one-dimensional consolidation under a given vertical stress. Accordingly, the two-stage behavior in cyclic loading is comparable to the primary and secondary consolidation. Such an observation is also found in RMF. Further investigation is required to provide solid evidence for the presence of two-stage behavior in other clayey soils.

## 5. Conclusions

Cyclic triaxial tests were carried out to investigate the dynamic properties of red mudstone fill material (RMF) under various cyclic stress levels and water contents. The dynamic state boundary surface (DSBS) was established using multiple mechanical parameters. The results show that traditional deformation indicators fail to clearly identify the shakedown and creep limits of RMF. Instead, dissipated energy serves as a reliable indicator: two threshold values of 0.01 kPa and 0.04 kPa are proposed as the unified shakedown and creep limits of RMF, independent of water content and cyclic stress. Relevant field tests have verified the effectiveness of this evaluation method.

Unlike the static state boundary surface (SBS), the DSBS accounts for the effect of loading cycles. A total of 50,000 loading cycles were adopted to capture the typical two-stage evolution of RMF in terms of permanent strain, equivalent Young’s modulus and dissipated energy. This two-stage characteristic remains stable even under a greater number of loading cycles. Further tests involving more loading cycles are required to explore the long-term evolution of the upper boundary of DSBS for permanent deformation.

Defining the elastic boundary is an important direction for improving the theoretical framework of the DSBS. Although viscosity leads to hysteresis at ultra-small strains, linear elasticity theory can roughly estimate the elastic limit of RMF. The established DSBS is applicable to practical engineering under train-load conditions at strains above 10^−4^. Introducing nonlinear dynamic elasticity theory will enhance the performance of the DSBS in analyzing behavior under small strains below 10^−4^.

## Figures and Tables

**Table 1:** Basic properties of RMF.

Atterberg Limits: %	Proctor Compaction Test	Particle Distribution: %	Specific Gravity *G*_s_	Mineralogical Composition: % (in Mass)
Liquid limit (LL): 32.5, Plastic limit (PL): 18.2, Plasticity index (PI): 14.3	Maximum dry density: 2.01 g/cm^3^, Optimal water content: 8.7%	Clay: 2.1, Silt: 89.6, Sand: 89.3	2.69	Quartz: 72, Smectite: 2, non-active minerals: 26

**Table 2:** Details of cyclic triaxial tests.

Initial Water Content, *w*_0_/%	Shear Strength, *q_f_*/kPa	*σ_s_*/*q_f_*	Cyclic Stress, $\sigma_{d}$	Loading Cycles, *N*	Variation in Water Content/%
5	972	0.46	150	50,000	−0.18
5	972	0.46	200	50,000	0.1
5	972	0.46	250	50,000	−0.06
5	972	0.46	300	50,000	−0.13
5	972	0.46	350	50,000	0.2
5	972	0.46	400	50,000	0.12
5	972	0.46	450	50,000	0.14
5	972	0.51	500	Failure at *N* < 1000	Not measured
7	899	0.50	100	50,000	−0.14
7	899	0.50	200	50,000	0.02
7	899	0.50	250	50,000	0.01
7	899	0.50	300	50,000	0.01
7	899	0.50	350	50,000	−0.13
7	899	0.50	400	50,000	0.19
7	899	0.50	450	50,000	0.15
7	899	0.50	500	Failure at *N* < 1000	Not measured
8.7	825	0.48	100	50,000	0.07
8.7	825	0.48	200	50,000	−0.14
8.7	825	0.48	250	50,000	0.27
8.7	825	0.48	300	50,000	−0.11
8.7	825	0.48	350	50,000	0.25
8.7	825	0.48	400	50,000	0.13
8.7	825	0.54	450	Failure at *N* < 1000	Not measured
11	688	0.51	80	50,000	0.34
11	688	0.51	100	50,000	0.26
11	688	0.51	120	50,000	0.15
11	688	0.51	140	50,000	−0.28
11	688	0.51	160	50,000	0.38
11	688	0.51	180	50,000	−0.13
11	688	0.51	220	50,000	−0.16
11	688	0.51	260	50,000	0.38
11	688	0.51	290	Failure at *N* < 2000	Not measured
13	581	0.52	80	50,000	0.58
13	581	0.52	100	50,000	0.47
13	581	0.52	120	50,000	−0.12
13	581	0.52	140	50,000	0.18
13	581	0.52	160	50,000	0.27
13	581	0.52	180	50,000	−0.38
13	581	0.52	220	50,000	0.28
13	581	0.52	260	Failure at *N* < 2000	Not measured

## Data Availability

The original contributions presented in this study are included in the article. Further inquiries can be directed to the corresponding author.

## Abbreviations

RMF	red mudstone fill material
UGMs	unbound granular materials
SBS	state boundary surface
DSBS	dynamic state boundary surface
*e*_0_	initial void ratio
*w*_0_	initial water content
*G_s_*	specific gravity
*σ*_3_	confined pressure
*σ* *_s_*	static mean deviator stress
*σ* *_d_*	cyclic stress
*σ* *_d__e_*	cyclic stress on elastic boundary
*σ* *_dnt_*	maximum cyclic stress generated in top layer of the subgrade induced by the specific axis train load
*q_f_*	shear strength
*v_q_*	loading rate of static mean deviator stress
*N*	loading cycles
*ε* *_ap_*	permanent axial strain
*ε_ample_*	axial strain amplitude
*v_ap_*	axial strain rate
*E_eq_*	equivalent Young’s modulus
*E_d_*	dissipated energy
*E_de_*	dissipated energy on elastic boundary
*E_dlA_*	shakedown limit
*E_dlB_*	creep limit
